# Interference between surgical magnetic drapes and pacemakers: an observational study comparing commercially available devices and a new magnetically isolated drape

**DOI:** 10.1186/s12938-016-0205-y

**Published:** 2016-07-19

**Authors:** Valerie Zaphiratos, Hubert Chiasson, Pierre Drolet, Bruno Benzaquen, Jacques Lapointe, Louis-Philippe Fortier

**Affiliations:** Department of Anesthesia, Maisonneuve-Rosemont Hospital, University of Montreal, 5415 Boul. de L’Assomption, Montreal, QC H1T 2M4 Canada; Department of Anesthesia, Grand-Portage regional Hospital, Riviere-du-Loup, QC Canada; Department of Cardiology, Maisonneuve-Rosemont Hospital, University of Montreal, Montreal, QC Canada

**Keywords:** Pacemaker, CIED, Magnet, Interference, Drape, Mat

## Abstract

**Background:**

Magnetic fields may potentially interfere with the function of cardiovascular implantable electronic devices. Sterile magnetic drapes used to hold surgical instruments are often placed on the patient’s thorax, and they are likely to interfere with the function of these devices.

**Methods:**

Thirty patients were recruited to compare a new prototype surgical magnetic drape (LT10G™ by Menodys) made with bottom-isolated ferrite magnets to the Covidien magnetic drape we used in a previous study. Twenty additional patients were recruited to compare the prototype drape with four commercially available surgical magnetic drapes.

**Results:**

Magnetic interference was found in 33 of the 50 total patients (70 %) when the Covidien drape was placed over the pacemaker. Of the 20 additional patients, 5 patients (25 %) displayed magnetic interference with a second type of surgical magnetic drape. A third magnetic drape caused interference in one patient (5 %), whereas a larger drape of the same model did not interfere in any patient. No patients demonstrated magnetic interference with the prototype drape.

**Conclusion:**

Bottom isolation of magnets in the prototype magnetic drape (LT10G™) used during surgery prevents magnetic interference in all patients when placed over the pacemaker. Three of the four commercially available magnetic drapes tested demonstrated magnetic interference. Flipping the prototype drape is not recommended as it may expose non-isolated magnets to the cardiovascular implantable electronic device.

**Electronic supplementary material:**

The online version of this article (doi:10.1186/s12938-016-0205-y) contains supplementary material, which is available to authorized users.

## Background

Over 1 million pacemakers and over 320,000 implantable cardioverter-defibrillators (ICDs) were implanted worldwide in 2009, and the implantation of cardiovascular implantable electronic devices (CIEDs) is on the rise [1–3]. Placing a magnet on a cardiac pacemaker usually enables an asynchronous mode in which the heart is paced at a predetermined frequency [4–7]. However, this magnet-activated asynchronous mode may have undesirable consequences, such as battery depletion, undesirable hemodynamic effects due to rapid pacing in some pacemakers, and rarely, ventricular dysrhythmias if pacemaker stimulation occurs in the vulnerable part of the cardiac cycle in patients with an intrinsic cardiac rhythm [4, 8–12]. In patients with an ICD, application of a magnet does not enable asynchronous pacing, but rather may suppress the detection of arrhythmias and prevents the device from delivering the appropriate therapy [4–6, 11, 12].

Sterile magnetic drapes are commonly used during surgery to hold metal instruments in the sterile field. The drape is often placed on the patient’s thorax, the usual location for a CIED. The placement of a magnetic drape over a pacemaker has been reported to result in unintended tachycardia and cardiac arrest [13]. In a previous study [14], it was found that surgical magnetic drapes are likely to activate the magnetic switch and cause asynchronous pacing in most patients when placed over the pacemaker. The magnetic drape tested in that study contained 70 ceramic ferrite magnets (116 Reusable Drape #31140588, Devon by Covidien, Mansfield, MA, USA). According to manufacturer’s specifications, over 5–10 Gauss is required to enable the asynchronous mode in the pacemakers tested in that study [14], a level reached when one of the 70 ferrite magnets from the drape is 3.4 cm away from the generator. There are several ways to reduce the magnetism of the drape, including alternating the polarities of adjacent magnets, or adding an isolating material between the magnet and the pacemaker.

Therefore, the purpose of this study was twofold. First, to compare the interference generated by the magnetic drape from Covidien used in a previous study [14] with a prototype magnetic drape manufactured with bottom-isolated ferrite magnets, currently commercialized as the LT10G™ by Menodys since September 2014, in order to determine in patients with a cardiac pacemaker whether asynchronous mode behavior develops. The second part of our study compares four different commercially available surgical magnetic drapes with the prototype drape containing bottom-isolated ferrite magnets. Our hypothesis is that placement of commercially available surgical magnetic drapes may result in asynchronous pacing in pacemaker patients, whereas the prototype magnetic drape with bottom-isolated ferrite magnets will not cause asynchronous pacing in these same patients.

## Methods

Following approval of the research ethics committee of the Maisonneuve-Rosemont Hospital affiliated to the University of Montreal, patients with an implanted cardiac pacemaker (Medtronic, Minneapolis, Minnesota, and Boston Scientific, Natick, MA, USA) were recruited for this study from October 2011 until November 2011 during regular device follow-up visits at the outpatient pacemaker clinic. After the cardiologist performed the initial scheduled device interrogation, the protocol was explained to all patients, and those who agreed to participate in the study, signed a written consent form.

Patient data including age, sex, height, weight, body mass index (BMI) were recorded. The protocol was performed with the patient supine, wearing a hospital gown. Continuous 3-lead electrocardiographic (ECG) monitoring was placed on the patient and the ECG was analysed by a medical Doctor (MD), an anesthesiologist or an anesthesiology senior resident, as well as a registered Nurse (RN) throughout the study. First, a strip of the patient’s baseline rhythm was obtained. Then, a Medtronic round magnet (#174105-2, Minneapolis, MN, USA) was placed on the pacemaker and a copy of the ECG to confirm the magnet mode behavior as specified by the manufacturer was obtained. Patients with ICDs and patients in whom the magnet rate cardiac rhythm was indistinguishable from their baseline rhythm were excluded. The round magnet was then removed.

**Fig. 1:**
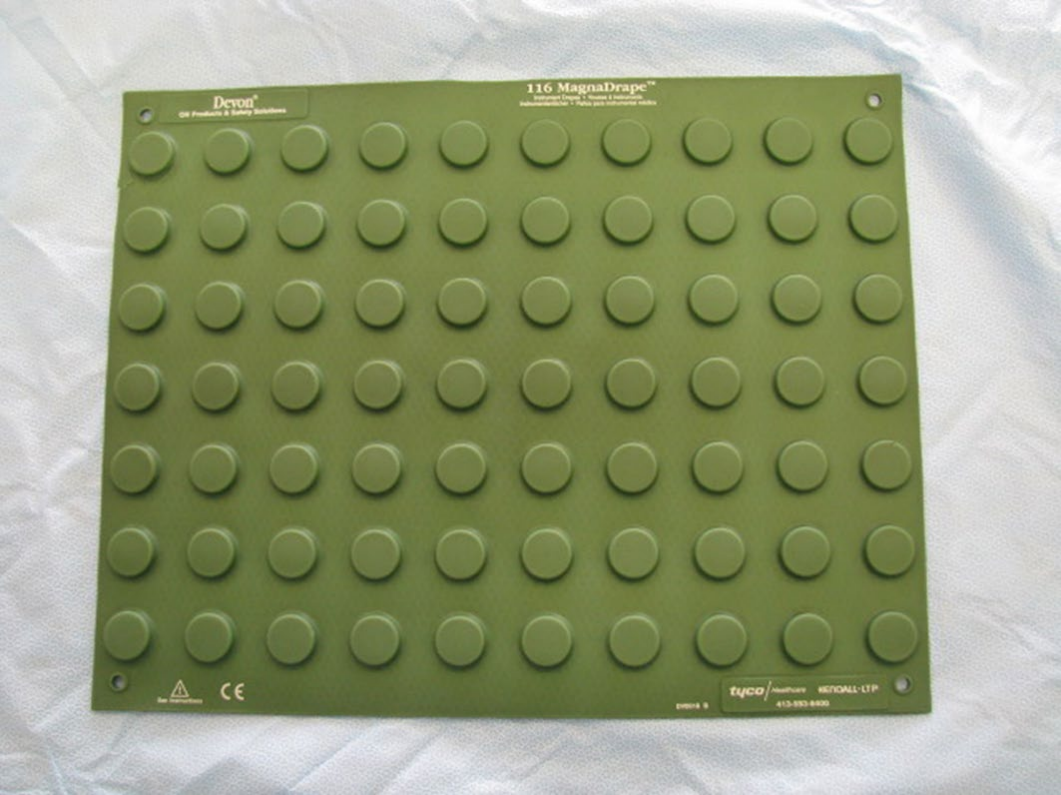
CVD surgical magnetic drape containing 70 ceramic ferrite magnets

**Fig. 2:**
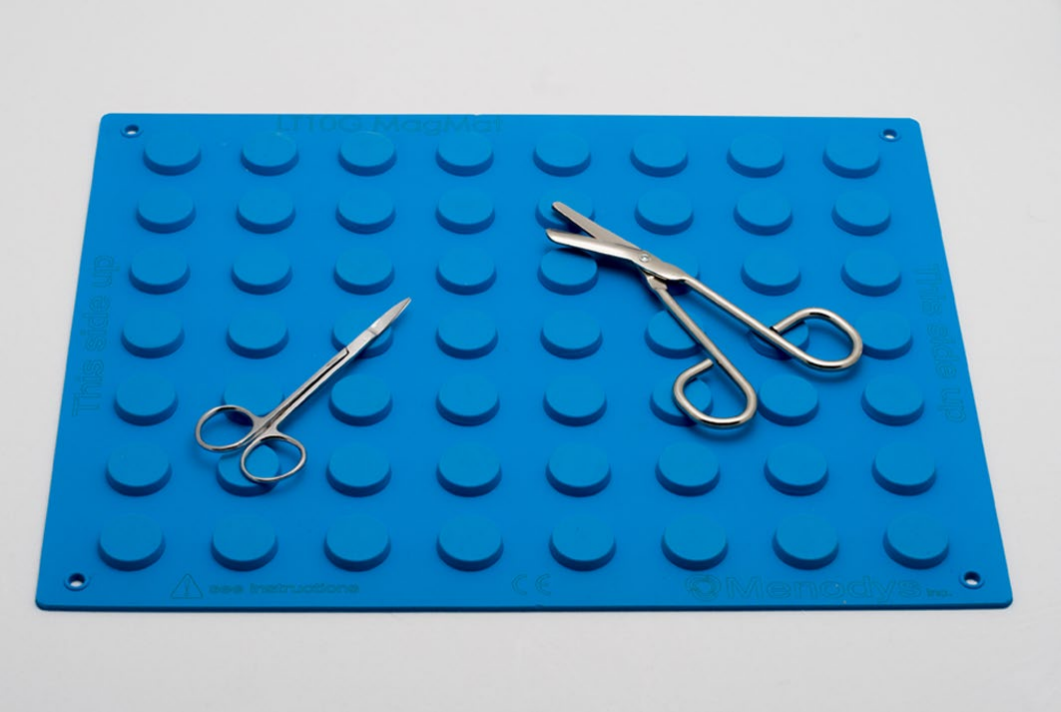
Prototype surgical magnetic drape with a silicone shell similar to the CVD drape, but instead contains 70 bottom-isolated ceramic ferrite magnets

**Fig. 3:**
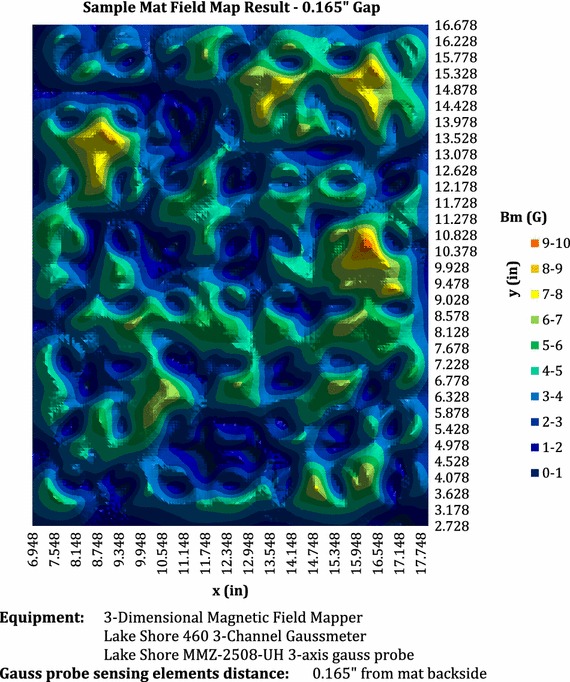
Magnetic field of the prototype drape on the patient facing side demonstrating an overall magnetic remanence less than 10 Gauss as measured at a distance of 0.165″ from the drape

Part 1 of the study was to assess two different magnetic drapes. A member of the personnel not involved in the study concealed the two magnetic drapes in an opaque plastic bag. The first drape (“CVD”) measured 29.5 cm × 37.5 cm and contained 70 ceramic ferrite magnets (116 Reusable Drape #31140588, Devon by Covidien, Mansfield, MA, USA) (Fig. 1). In a previous study [14], the magnets in the CVD drape were found to be inserted in a random fashion, regardless of polarity. In a single drape, the polarity of each magnet can impact the overall vector of magnetism of that drape. Thus, at the beginning of each data collection day, a different CVD drape was used in order to reduce bias caused by the potential magnetic signature of each commercial drape. The second magnetic drape was a prototype made with a silicone shell similar to the CVD drape, but instead contained 70 isolated ferrite magnets (Fig. 2). The isolation material consisted of a steel-based metal cup that diminished the magnetic remanence on the patient side of the drape and only encapsulated the underside of each ferrite magnet in order to create a unidirectional magnetic field. The overall magnetic field on the patient facing side was less than 10 Gauss (Fig. 3) (Additional file 1).

Each magnetic drape was concealed and centered over the patient’s pacemaker, with the help of a measuring tape, one after the other. The investigator performing the study and the data collector were both blinded to the type of magnetic drape. Magnetic interference was identified if the cardiac rhythm was asynchronous and identical to that produced by the round magnet rhythm. If there was no change in rhythm with the magnetic drape, the drape was displaced in 1–2 cm increments over the pacemaker in an effort to elicit an asynchronous rhythm. Once the protocol was completed, the patient was referred to the cardiologist who, if the situation permitted, discharged the patient.

Part 2 of the study consisted of evaluating and comparing 4 different commercially available magnetic drapes, including the CVD, with the prototype magnetic drape. All four of these commercially available magnetic drapes underwent the same protocol as the magnetic drapes in part 1 of the study. Due to the different sizes and shapes of the four drapes, it was not possible to create a blinded evaluation with an opaque plastic bag. The magnetic drapes evaluated were the CVD magnetic drape, the “JCM” magnetic drape (Reusable Magnetic Pad, Jac-Cell Medic, Town of Mount-Royal, Quebec, Canada), the “LDR” magnetic drape (Magnetic Instrument Pad #25-002, size 20″ × 16″, DeRoyal Industries Inc., Powell, TN, USA), and the “SDR” magnetic drape (Magnetic Instrument Pad #25-001, size 10″ × 16″, DeRoyal Industries Inc., Powell, TN, USA).

The sample size calculation for the primary outcome (first part of the study) was based on the direct comparison between the proportion of patients experiencing pacemaker interference associated with the CVD drape in one group and the new prototype in the other. A previous study having shown that using the CVD drape caused pacemaker interference in 70 % of participants [14], we elected to look for a 50 % reduction (from 70 to 35 %) in interference with the prototype drape. It was then calculated that 30 patients per group were needed to show such a difference with alpha and beta errors of 0.05 and 0.8 respectively.

The sample size calculation for part 2 was to be derived from the results obtained during the first part of the study, using the proportion of patients experiencing interference with the prototype as a base for calculation. Since no case of interference was observed, it was arbitrarily decided to recruit twenty participants to provide an exploratory overview of the pacemaker interference associated with the various commercially available devices.

Fisher’s exact test was used for comparisons in the first part of the study. Only descriptive statistics were used for the second part. Calculations and analyses were performed with Prism 5.0 statistical package (GraphPad Software Inc, La Jolla, CA, USA). Unless stated otherwise, data are presented as mean ± SD and a *p* value <0.05 was deemed significant.

## Results

Demographic criteria of the patients for both parts of the study are listed in Table 1. Thirty patients were recruited in part 1, and three were excluded due to a clerical mistake in the make and model of magnetic drape that had been placed in the opaque plastic bag. Of the 27 patients studied, 17 had a Boston Scientific pacemaker and 10 patients had a Medtronic pacemaker. All pacemakers were placed subcutaneously. Seventeen patients (63 %) displayed asynchronous cardiac pacing when the CVD drape was placed over the pacemaker, whereas none of the patients displayed magnetic interference with the prototype drape (*p* < 0.0001) (Table 2).

**Table 1:** Demographic data

Demographic	Part 1	Part 2
Patients	N = 27	N = 20
Gender	M 15:F 12	M 10:F 10
Age (years) (mean ± SD)	79 ± 8	78 ± 8
Height (m) (mean ± SD)	1.67 ± 0.07	1.66 ± 0.09
Weight (kg) (mean ± SD)	75 ± 17.6	73 ± 16
Body mass index (kg/m^2^) (mean ± SD)	27 ± 5.2	27 ± 4
Type of pacemaker (Boston Scientific/Medtronic)	17/10	9/11

*SD* standard deviation

**Table 2:** Results from Part 1 of the study (n = 27)

	Asynchronous rhythm	
	CVD drape	Prototype drape
Patients (%)	N = 17 (63 %)	N = 0
Age (years) (mean ± SD)	81 ± 6	N/A
Gender	M 11/F 6	N/A
Weight (kg) (mean ± SD)	74 ± 18	N/A
Height (m) (mean ± SD)	1.68 ± 0.07	N/A
Body mass index (kg/m^2^) (mean ± SD)	26 ± 5	N/A
Pacemaker (Boston Scientific/Medtronic)	7/10	N/A

In part 2 of the study, 20 patients were recruited and studied. Nine had a Boston Scientific pacemaker and 11 patients had a Medtronic pacemaker. All pacemakers were placed subcutaneously. Sixteen patients (80 %) demonstrated asynchronous cardiac pacing with the CVD drape. For the JCM magnetic drape, 5 patients (25 %) displayed asynchronous cardiac pacing. The LDR magnetic drape did not cause asynchronous cardiac pacing in any patient. The unfolded SDR drape displayed asynchronous cardiac pacing in 1 patient (5 %). No patients demonstrated asynchronous cardiac pacing with the prototype drape. No patients exhibited any adverse outcomes during the study (Table 3). Detailed results for each patient are available in Appendix.

**Table 3:** Results from part 2 of the study (n = 20)

Drapes tested	Prototype	CVD	JCM	SDR	LDR
Patients who demonstrated asynchronous rhythm (%)	N = 0	N = 16 (80 %)	N = 5 (25 %)	N = 1 (5 %)	N = 0
Age (years) (mean ± SD)	N/A	80 ± 7	82 ± 7	87	N/A
Gender	N/A	M 7/F 9	M 4/F 1	M 0/F 1	N/A
Weight (kg) (mean ± SD)	N/A	69 ± 15	70 ± 18	44	N/A
Height (m) (mean ± SD)	N/A	1.66 ± 0.09	1.74 ± 0.08	1.52	N/A
Body mass index (kg/m^2^) (mean ± SD)	N/A	26 ± 4	26 ± 3	19	N/A
Pacemaker (Boston Scientific/Medtronic)	N/A	6/10	3/2	0/1	N/A

## Discussion

The findings in this study suggest that bottom-isolation of the magnets in an unfolded magnetic drape used during surgery prevents asynchronous pacing in all patients when placed over the pacemaker. Three of the four commercially available magnetic drapes tested demonstrated asynchronous cardiac pacing.

For both parts of this study, the CVD drape caused asynchronous pacing in a total of 33 patients (70 %). These results mirror a previous study [14] where 70 % of patients demonstrated asynchronous rhythm with the CVD magnetic drape. Although the JCM magnetic drape caused asynchronous pacing in 25 % of patients, this is less than that of the CVD magnetic drape. This is possibly due to the fact that the magnets of the JCM drape are placed further apart creating a weaker magnetic field compared to those in the CVD. Since we did not test the remanence of the individual magnets in each of the commercially available magnetic drapes in this study, we cannot postulate on the strength of their remanence compared to the magnets in the CVD.

Among the other commercially available drapes, the LDR magnetic drape did not exhibit magnetic pacemaker interference. Considering the LDR magnetic drape is of the same make and model as the SDR drape, but simply 10″ larger, it is possible that our sample size of 20 patients was not adequate to elicit asynchronous rhythm with the LDR drape. In a previous study [14], it was suggested that a single well-positioned magnet can induce asynchronous rhythm. Thus, it is possible that the position of the magnets in the SDR drape we tested created a stronger magnetic field than those of that particular LDR drape. This may no longer be true when switching for another identical LDR or SDR drape of the same make and model. Unfortunately, we did not test for this in our study. Contrary to the CVD and JCM drapes, the LDR and SDR drapes are disposable, and it is possible that the remanence of the disposable drapes may be lesser or may have diminished during the study period, as they are meant for single use only. From our results, it is reasonable to propose that at least a subset of the magnetic drapes produced by this company may generate magnetic interference with pacemakers.

The prototype magnetic drape, currently commercialized as the LT10G™ by Menodys, is manufactured to diminish the magnetic remanence on the patient facing side of the drape. This is achieved by having each individual magnet of the drape encapsulated in a cup with a steel-based metal isolation material. Due to this technology, no patient in our study demonstrated an asynchronous cardiac rhythm with the LT10G™ magnetic drape.

## Limitations

Our study was limited to the magnetic drapes and pacemakers tested. Thus, our results may not apply to all commercially available magnetic drapes and pacemakers. In addition, our sample size for the second part of our study was arbitrarily determined and may warrant further investigation with more patients and different types of magnetic drapes and pacemakers.

Few studies have described the risk of interference between magnets and CIEDs, and only one study has specifically examined magnetic surgical drapes and it demonstrated that the unfolded CVD magnetic drape caused asynchronous rhythm in 70 % of patients [14]. In addition, the authors describe that magnetic interference decreases markedly with increasing caudal distance from the pacemaker. Other studies describe the risk of interference between magnets and CIEDs. Ryf et al. [15]. demonstrated in an in vitro study that different neodymium magnets found in everyday life, such as in toys and jewelry can all activate asynchronous pacing modes in pacemakers when placed at various distances from the device. Magnetic interference with different neodymium magnets occurred when placed up to 3 cm to the device [16]. Prolonged exposure to similar magnets were implicated in intermittent, erratic behavior of an ICD [17] and deactivation of an ICD resulting in a fatal consequence [18]. In a clinical study by Hiller et al. [19], three of 12 patients with a pacemaker experienced interference by small dental mini-magnets. The effect disappeared once the magnets were pulled 1 cm away.

## Conclusion

Commercially available surgical magnetic drapes may result in asynchronous pacing. Three of the four commercially available magnetic drapes tested demonstrated magnetic interference. The prototype magnetic drape with bottom-isolated ferrite magnets tested in our study did not cause asynchronous pacing in these same patients. This new technology has the potential to enhance the safety of the operating room environment for patients with CIEDs. Flipping the prototype drape is not recommended as it may expose non-isolated magnets to the CIED.

## Appendix

See Tables 4 and 5.

**Table 4:** Detailed results for each patient from part 1 of the study

Subject	Date	Age (years)	Gender	Weight (kg)	Height (m)	BMI	Pacemaker model	Asynchronous rhythm with CVD drape	Displacement^a^	Asynchronous rhythm with prototype drape (with and without displacement)
1	20/10/2011	82	F	68	1.68	24.02	Boston Scientific	No	–	No
2	20/10/2011	73	M	82	1.78	25.75	Boston Scientific	Yes	Yes	No
3	20/10/2011	81	F	60	1.60	23.44	Boston Scientific	No	–	No
4	20/10/2011	65	M	84	1.73	28.20	Boston Scientific	No	–	No
5	27/10/2011	82	M	66	1.75	21.49	Boston Scientific	No	–	No
6	27/10/2011	52	M	102	1.78	32.19	Boston Scientific	No	–	No
7	27/10/2011	78	F	54	1.63	20.47	Boston Scientific	Yes	Yes	No
8	27/10/2011	75	F	88	1.60	34.38	Boston Scientific	No	–	No
9	27/10/2011	84	F	59	1.60	23.05	Boston Scientific	No	–	No
10	27/10/2011	86	M	77	1.70	26.68	Boston Scientific	No	–	No
11	27/10/2011	82	M	95	1.73	31.84	Boston Scientific	Yes	No	No
12	27/10/2011	81	F	61	1.60	23.91	Boston Scientific	Yes	No	No
13	27/10/2011	70	F	55	1.52	23.81	Boston Scientific	No	–	No
14	27/10/2011	76	M	85	1.74	28.17	Boston Scientific	Yes	Yes	No
15	27/10/2011	76	M	56	1.64	20.82	Boston Scientific	Yes	No	No
16	27/10/2011	80	F	77	1.65	28.17	Boston Scientific	Yes	Yes	No
17	27/10/2011	73	F	102	1.65	37.50	Boston Scientific	No	–	No
18	3/11/2011	81	M	64	1.68	22.50	Medtronic	Yes	Yes	No
19	3/11/2011	92	M	57	1.70	19.62	Medtronic	Yes	Yes	No
20	3/11/2011	85	F	69	1.63	25.78	Medtronic	Yes	Yes	No
21	3/11/2011	75	M	94	1.75	30.79	Medtronic	Yes	Yes	No
22	3/11/2011	85	M	66	1.65	24.17	Medtronic	Yes	Yes	No
23	3/11/2011	77	M	93	1.65	34.31	Medtronic	Yes	Yes	No
24	3/11/2011	76	M	108	1.83	32.25	Medtronic	Yes	No	No
25	3/11/2011	90	M	61	1.68	21.68	Medtronic	Yes	Yes	No
26	3/11/2011	87	F	45	1.55	18.90	Medtronic	Yes	Yes	No
27	3/11/2011	77	F	93	1.65	34.16	Medtronic	Yes	No	No

^a^Displacement was necessary in order to obtain asynchronous rhythm

**Table 5:** Detailed results for each patient for part 2 of the study

Subject	Age	Gender	Weight (kg)	Height (m)	BMI	Pacemaker model	Asynchronous rhythm with drape				
							Prototype	JCM	LDR	SDR	CVD
1	74	M	89	1.78	28.1	Medtronic	No	No	No	No	No
2	87	F	44	1.52	19	Medtronic	No	No	No	Yes	Yes
3	66	M	89	1.73	29.7	Medtronic	No	No	No	No	Yes
4	78	M	70	1.70	24.2	Medtronic	No	No	No	No	Yes
5	86	F	52	1.60	20.3	Medtronic	No	No	No	No	Yes
6	87	F	66	1.55	27.5	Medtronic	No	No	No	No	Yes
7	88	F	61	1.65	22.4	Medtronic	No	No	No	No	Yes
8	81	M	77	1.80	23.8	Medtronic	No	Yes	No	No	Yes
9	69	M	80	1.60	31.2	Boston Scientific	No	No	No	No	No
10	75	M	60	1,49	28.6	Boston Scientific	No	Yes	No	No	Yes
11	64	F	94	1.60	36.7	Boston Scientific	No	No	No	No	No
12	92	F	46	1,49	21.9	Boston Scientific	No	Yes	No	No	Yes
13	78	F	89	1.70	30.8	Boston Scientific	No	No	No	No	Yes
14	76	M	91	1.75	29.7	Boston Scientific	No	No	No	No	No
15	83	M	95	1.78	30	Boston Scientific	No	Yes	No	No	Yes
16	79	F	79	1.68	28	Boston Scientific	No	No	No	No	Yes
17	72	M	75	1.75	24.5	Medtronic	No	No	No	No	Yes
18	80	F	63	1.60	24.6	Boston Scientific	No	No	No	No	Yes
19	72	F	66	1.52	28.6	Medtronic	No	No	No	No	Yes
20	77	M	70	1.65	25.7	Medtronic	No	Yes	No	No	Yes

## Additional file

10.1186/s12938-016-0205-y Patent for the LT10G^TM^ drape from Menodys: US patent 9,232,976. Dated January 12th, 2016. http://patft.uspto.gov/netacgi/nph-Parser?Sect1=PTO1&Sect2=HITOFF&d=PALL&p=1&u=%2Fnetahtml%2FPTO%2Fsrchnum.htm&r=1&f=G&l=50&s1=9232976.PN.&OS=PN/9232976&RS=PN/9232976.

## Abbreviations

ICD: implantable cardioverter-defibrillator
CIED: cardiovascular implantable electronic device
BMI: body mass index
ECG: electrocardiogram
MD: Medical Doctor
RN: registered Nurse
CVD: Devon by Covidien drape
JCM: Jac-Cell medic drape
LDR: DeRoyal Industries Inc. drape, size 20″ × 16″
SDR: DeRoyal Industries Inc. drape, size 10″ × 16″
