# Supplementary material for: Interference between surgical magnetic drapes and pacemakers: an observational study comparing commercially available devices and a new magnetically isolated drape
Source: Biomed Eng Online. 2016 Jul 19;15:83. doi: 10.1186/s12938-016-0205-y (PMC4949887; doi:10.1186/s12938-016-0205-y)
Supplement: Supplementary file 1 — 10.1186/s12938-016-0205-y Patent for the LT10GTM drape from Menodys: US patent 9,232,976. Dated January 12th, 2016. http://patft.uspto.gov/netacgi/nph-Parser?Sect1=PTO1&Sect2=HITOFF&d=PALL&p=1&u=%2Fnetahtml%2FPTO%2Fsrchnum.htm&r=1&f=G&l=50&s1=9232976.PN.&OS=PN/9232976&RS=PN/9232976. [file 12938_2016_205_MOESM1_ESM.pdf]

US009232976B2

(12) United States Patent
Fortier et al.

(10) Patent No.: US 9,232,976 B2
(45) Date of Patent: Jan. 12, 2016

(54) MAGNETIC INTERFERENCE REDUCING SURGICAL DRAPE

(75) Inventors: **Louis-Philippe Fortier**, Anjou (CA); **Valérie Zaphiratos**, Montréal (CA); **Howard Burman**, Montréal (CA); **Daniel Spooner**, Lachine (CA); **René Gosselin**, Longueuil (CA); **Richard Côté**, Longueuil (CA)

(73) Assignee: **RSEM LIMITED PARTNERSHIP**, Montreal (CA)

(*) Notice: Subject to any disclaimer, the term of this patent is extended or adjusted under 35 U.S.C. 154(b) by 357 days.

(21) Appl. No.: **13/805,448**

(22) PCT Filed: **Jun. 23, 2011**

(86) PCT No.: **PCT/CA2011/050387**

§ 371 (c)(1),
(2), (4) Date: **Mar. 1, 2013**

(87) PCT Pub. No.: **WO2011/160236**

PCT Pub. Date: **Dec. 29, 2011**

(65) **Prior Publication Data**

US 2013/0199544 A1 Aug. 8, 2013

Related U.S. Application Data

(60) Provisional application No. 61/357,722, filed on Jun. 23, 2010, provisional application No. 61/434,986, filed on Jan. 21, 2011.

(51) **Int. Cl.**
A61B 19/08 (2006.01)
A61B 19/10 (2006.01)

(Continued)

(52) **U.S. Cl.**
CPC ***A61B 19/10*** (2013.01); ***A61C 19/00*** (2013.01); ***A61D 1/00*** (2013.01); ***H01F 7/0252*** (2013.01);

(Continued)

(58) **Field of Classification Search**
USPC 128/852
See application file for complete search history.

(56) **References Cited**

U.S. PATENT DOCUMENTS

3,483,494 A 12/1969 Cromie
3,727,658 A 4/1973 Eldridge, Jr.

(Continued)

FOREIGN PATENT DOCUMENTS

EP 0 081 225 A1 6/1983
WO 00/48526 A1 8/2000

(Continued)

Primary Examiner — Ophelia A Hawthorne
(74) *Attorney, Agent, or Firm* — Norton Rose Fulbright Canada LLP

(57) **ABSTRACT**

A magnetic drape comprises a drape body made of a flexible material. The drape body has a panel portion having an undersurface adapted to be laid on an uneven body and a top surface, and a given thickness between the undersurface and the top surface. A plurality of magnet units are within the flexible material of the drape body, with each said magnet unit comprising a shielding material in the shape of a cup oriented to have a bottom wall facing toward the undersurface of the drape body, and a magnet received at least partially in the cup. The magnet comprises two or more sections arranged to expose opposite polarity on a top surface of the magnetic drape, with each said section having opposite polarities oriented vertically.

19 Claims, 11 Drawing Sheets

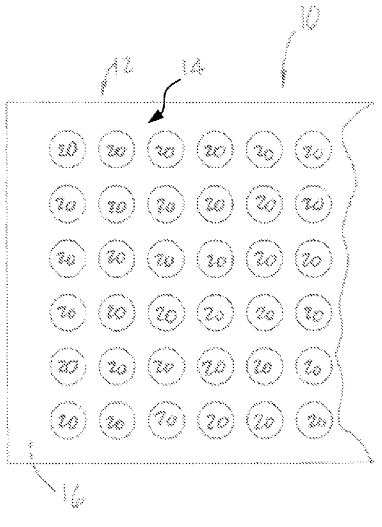

(51) **Int. Cl.**

A61C 19/00	(2006.01)
A61D 1/00	(2006.01)
H01F 7/02	(2006.01)
A61B 17/00	(2006.01)
A61B 19/00	(2006.01)

(52) **U.S. Cl.**

CPC *A61B 19/088* (2013.01); *A61B 2017/00876* (2013.01); *A61B 2019/4036* (2013.01); *A61B 2019/5441* (2013.01)

(56) **References Cited**

U.S. PATENT DOCUMENTS

4,185,262	A	1/1980	Watanabe	
4,373,629	A	2/1983	Ulin et al.	
4,524,767	A	6/1985	Glassman	
4,944,311	A *	7/1990	Eldridge et al.	128/849
5,195,538	A	3/1993	Eldridge	
5,538,495	A	7/1996	Ardizzone	
5,578,359	A *	11/1996	Forbes et al.	428/131
6,522,145	B1 *	2/2003	Damadian et al.	324/318
2002/0050311	A1	5/2002	Palumbo	
2002/0084008	A1	7/2002	Limoges et al.	
2002/0088515	A1	7/2002	Aust et al.	
2003/0234181	A1	12/2003	Palumbo	
2004/0112486	A1	6/2004	Aust et al.	
2005/0205425	A1	9/2005	Palumbo	
2006/0135281	A1	6/2006	Palumbo	
2006/0135282	A1	6/2006	Palumbo	
2006/0160636	A1	7/2006	Palumbo	
2006/0292388	A1	12/2006	Palumbo	
2007/0281176	A1	12/2007	Palumbo	
2008/0090066	A1	4/2008	Palumbo	
2008/0107805	A1	5/2008	Palumbo	
2008/0119307	A1	5/2008	Palumbo	
2008/0254310	A1	10/2008	Palumbo	
2009/0027149	A1	1/2009	Kocijan	
2009/0159451	A1	6/2009	Tomantschger et al.	
2009/0267717	A1 *	10/2009	Baskett	335/285
2009/0298624	A1	12/2009	Palumbo	
2010/0007449	A1	1/2010	Tait et al.	
2010/0028714	A1	2/2010	Palumbo	
2010/0076556	A1	3/2010	Tomantschger et al.	
2010/0239801	A1	9/2010	Elia et al.	
2010/0291381	A1	11/2010	Elia et al.	
2010/0304063	A1	12/2010	McCrea et al.	
2010/0304065	A1	12/2010	Tomantschger et al.	
2010/0304171	A1	12/2010	Tomantschger et al.	
2010/0304172	A1	12/2010	Facchini et al.	
2010/0304179	A1	12/2010	Facchini et al.	
2010/0304182	A1	12/2010	Facchini et al.	
2010/0307642	A1	12/2010	Palumbo	
2011/0003171	A1	1/2011	Palumbo	

FOREIGN PATENT DOCUMENTS

WO	20040105528	A2	9/2004
WO	20080141137	A2	11/2008

* cited by examiner

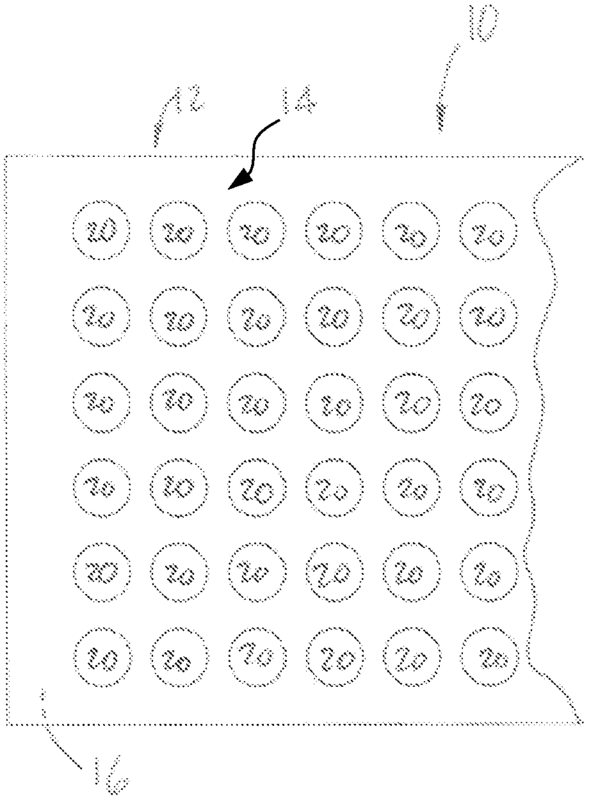

Fig. 1

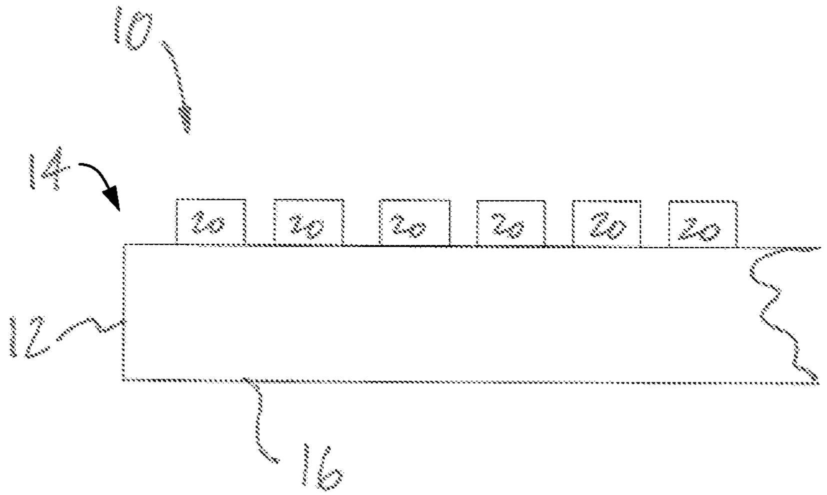

Fig. 2

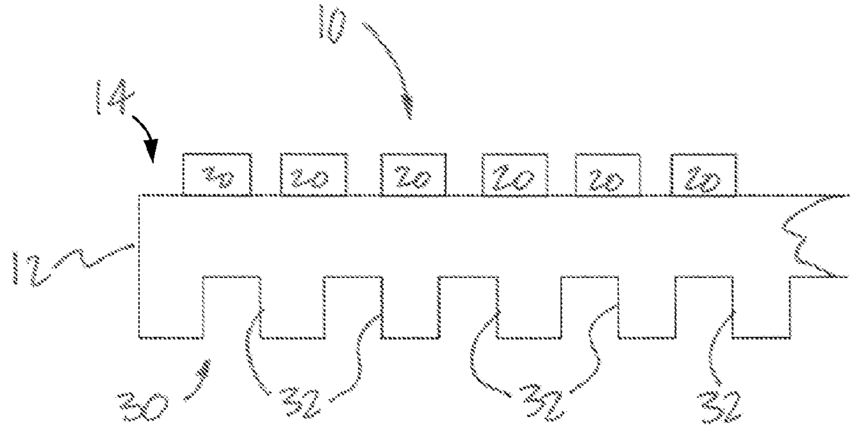

Fig. 3

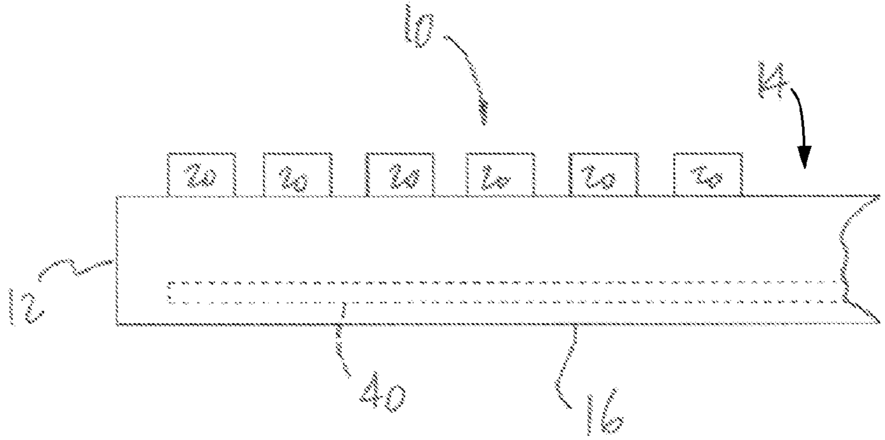

Fig. 4

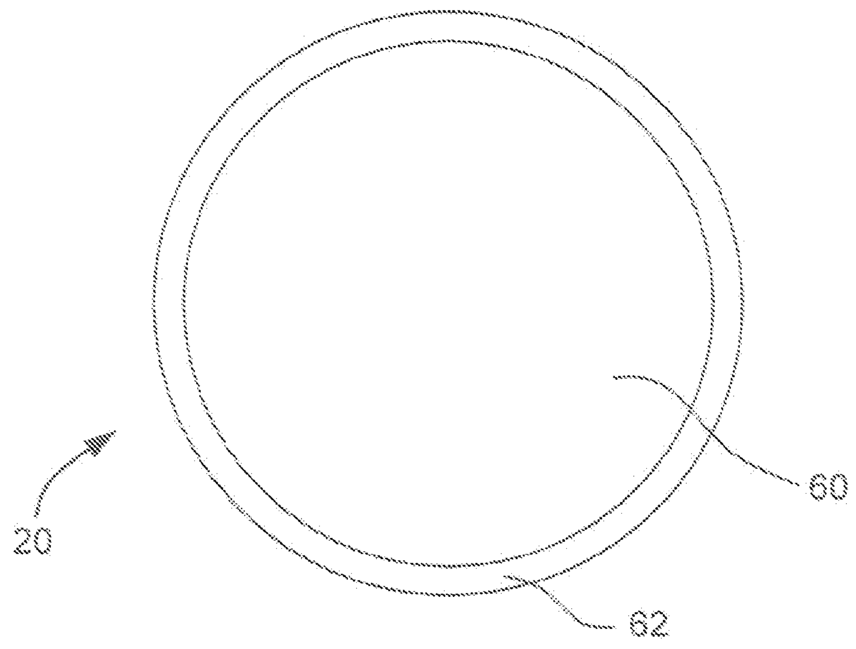

Fig. 5A

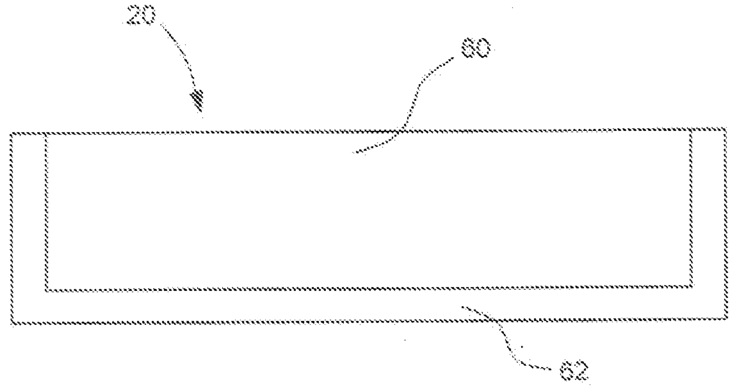

Fig. 5B

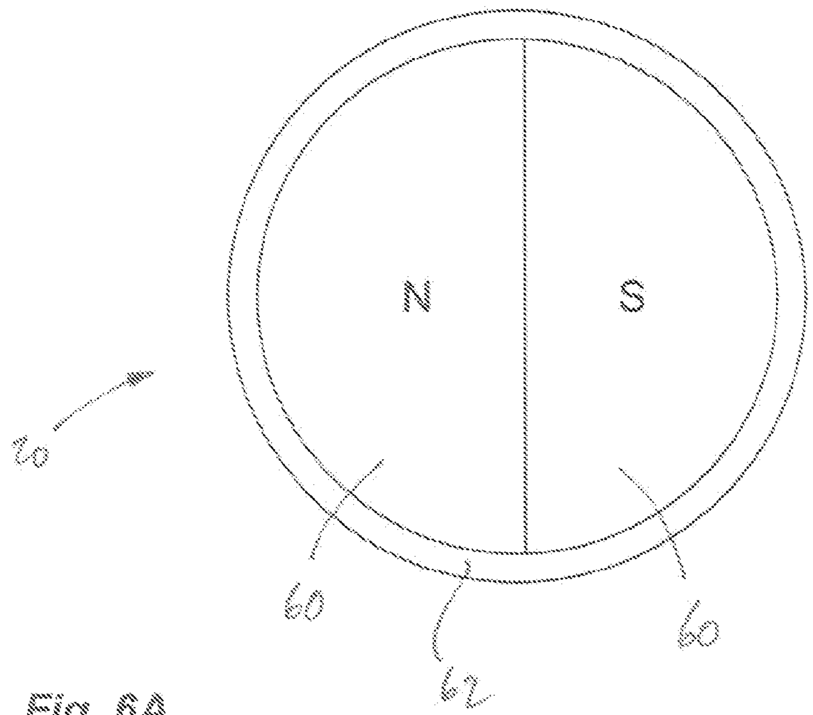

Fig. 6A

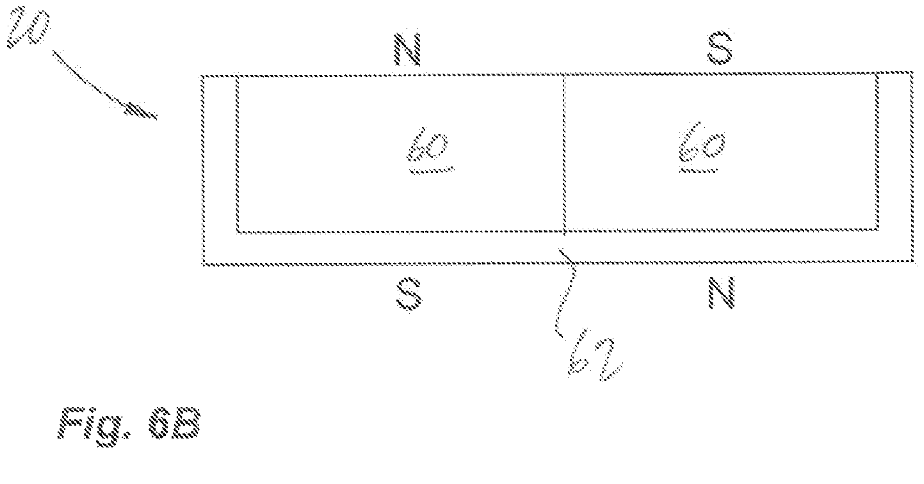

Fig. 6B

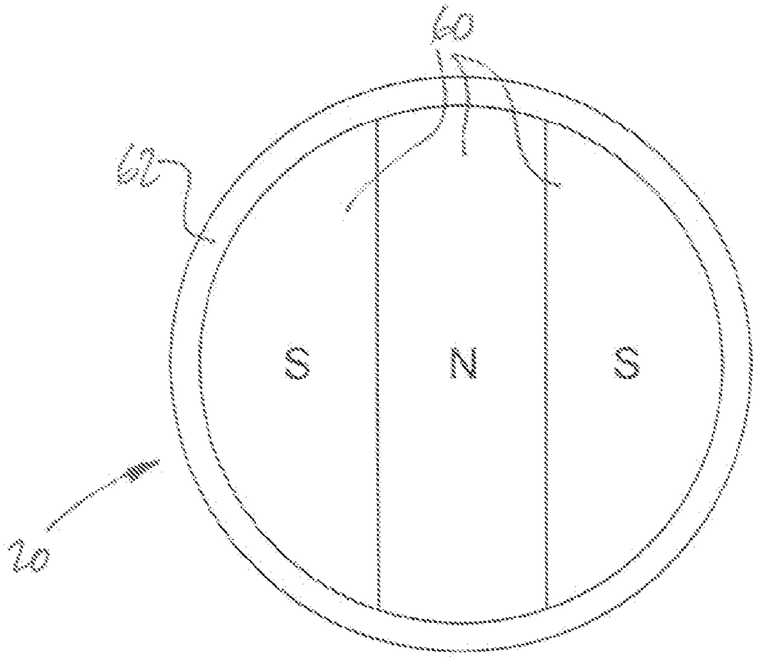

Fig. 7

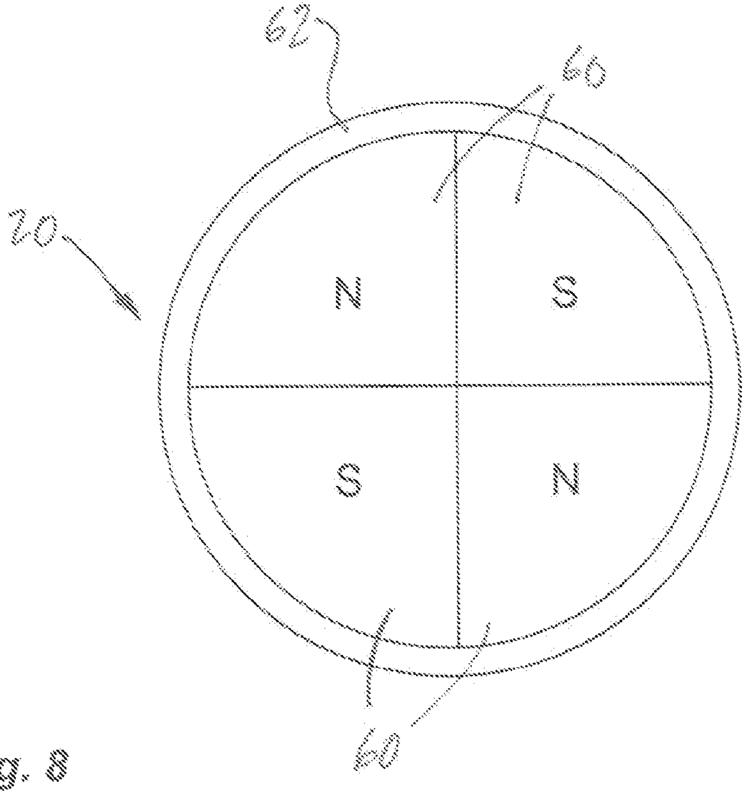

Fig. 8

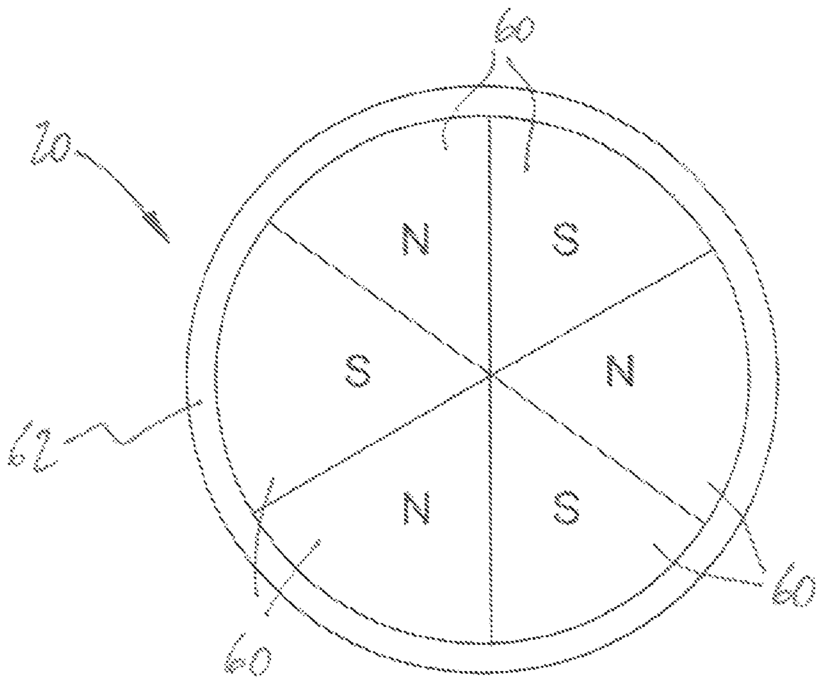

Fig. 9

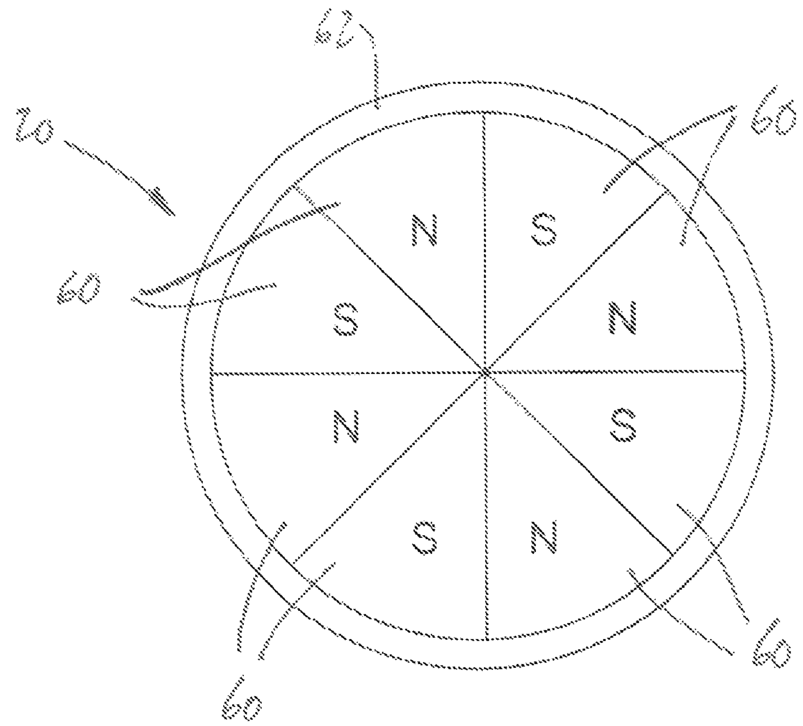

Fig. 10

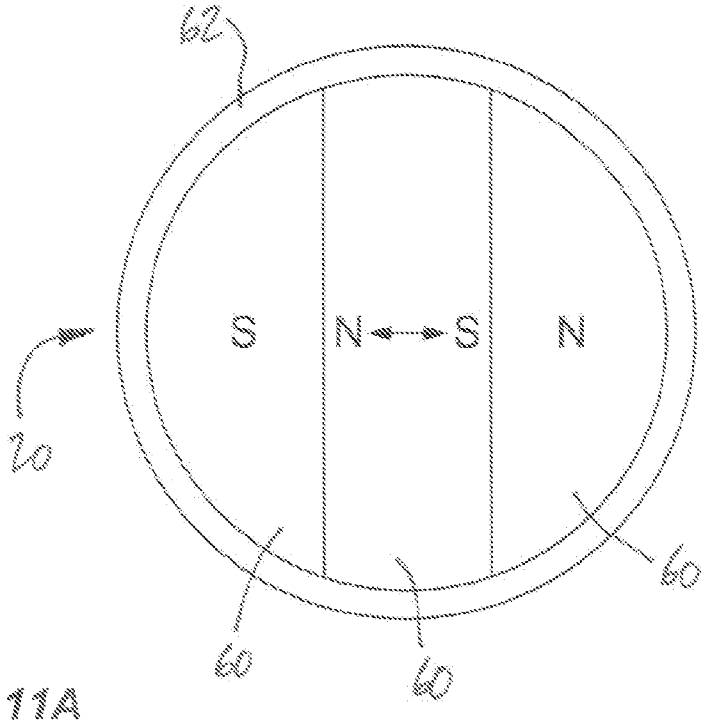

Fig. 11A

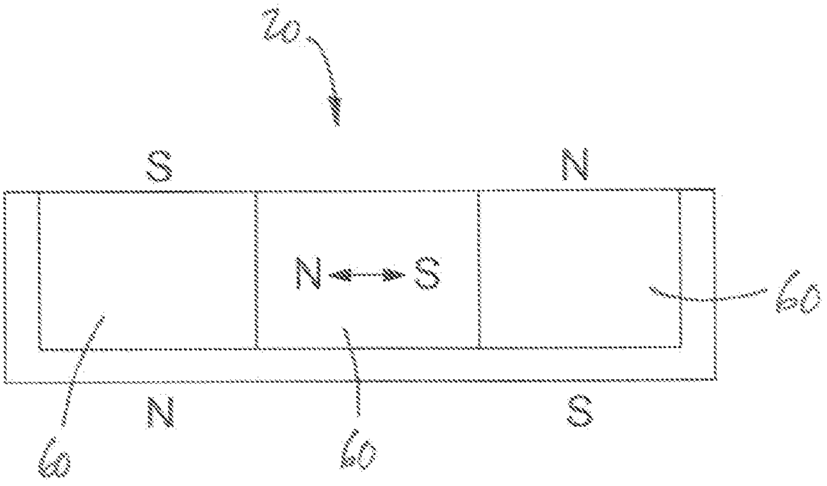

Fig. 11B

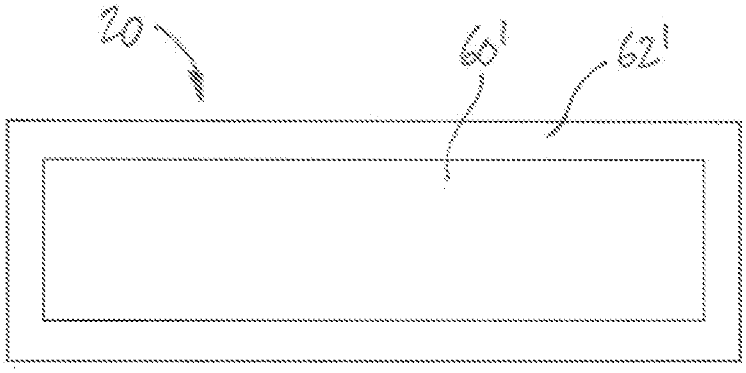

Fig. 12A

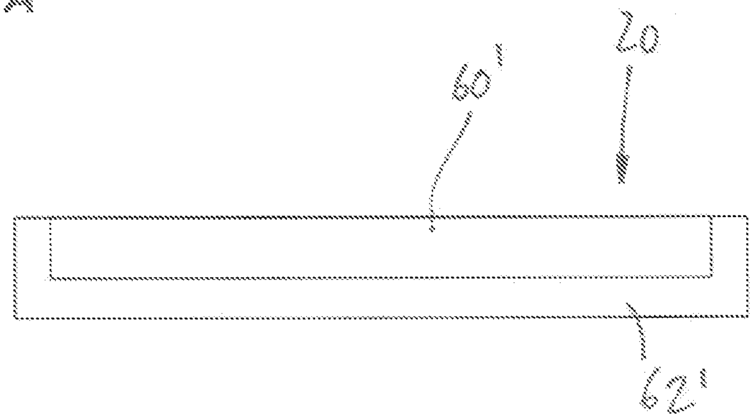

Fig. 12B

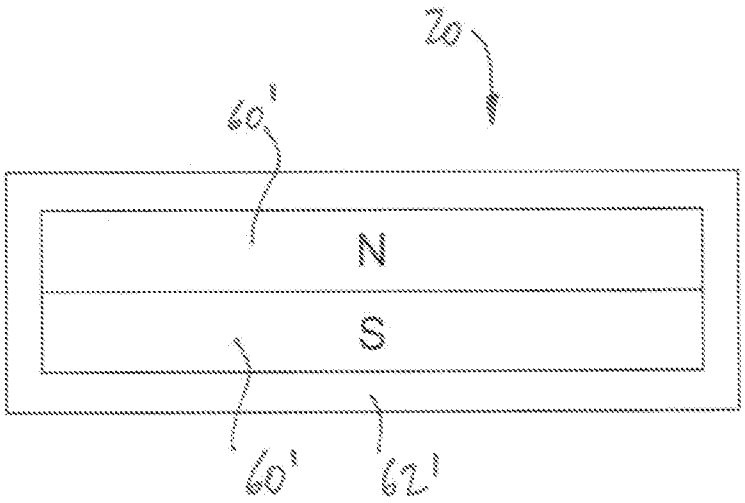

Fig. 13

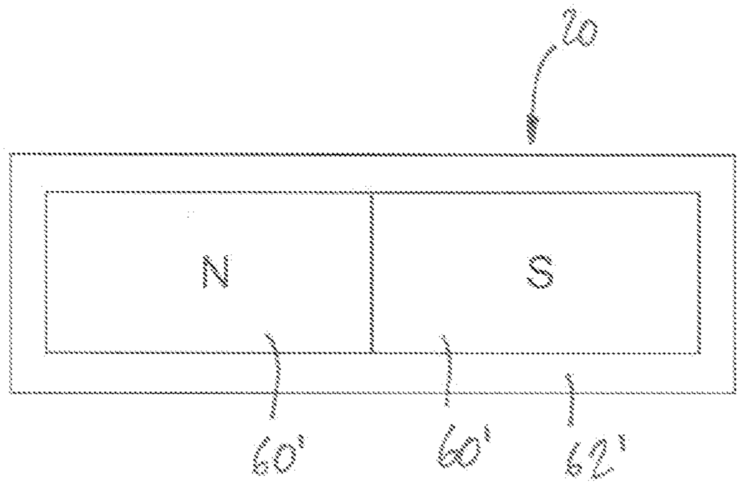

Fig. 14A

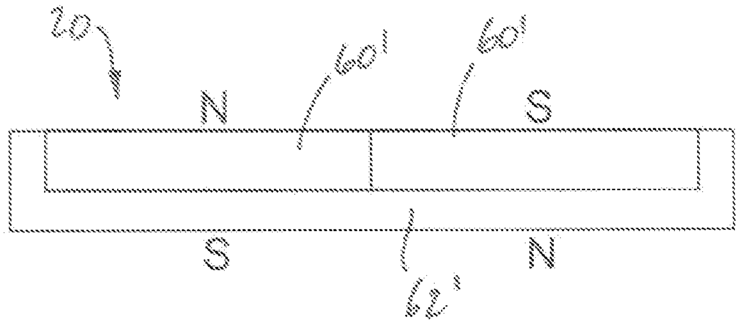

Fig. 14B

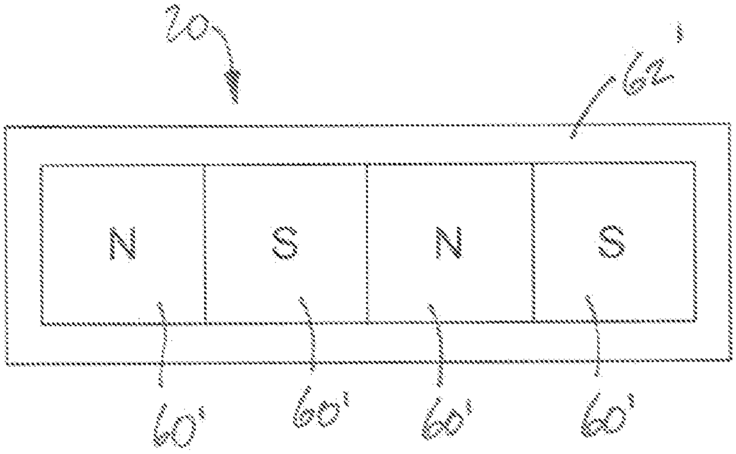

Fig. 15

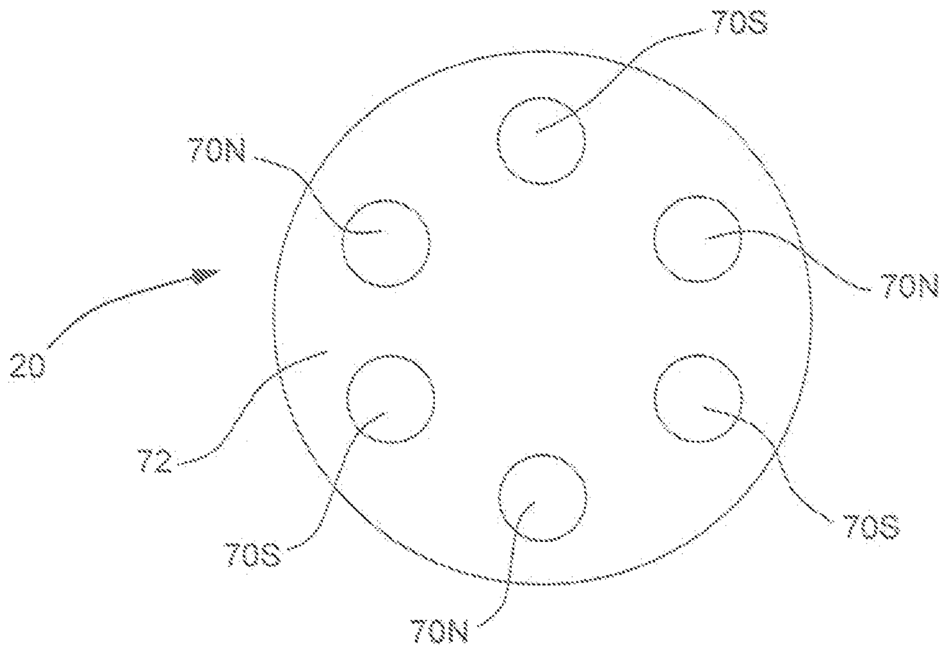

Fig. 16

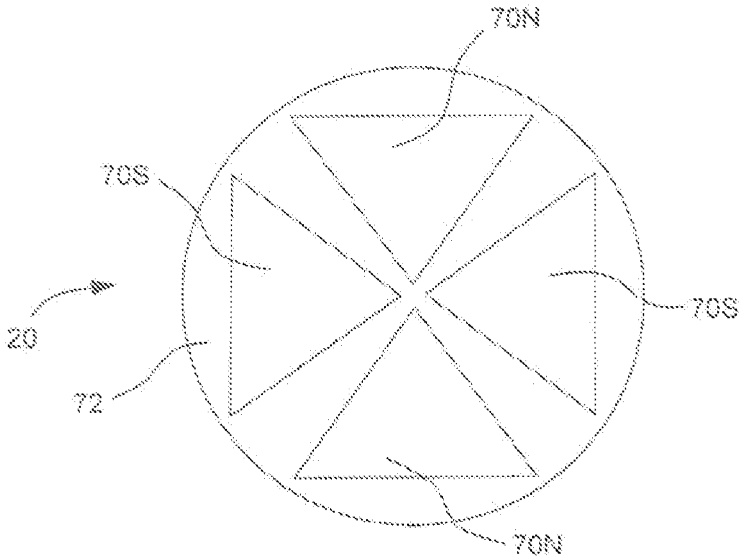

Fig. 17

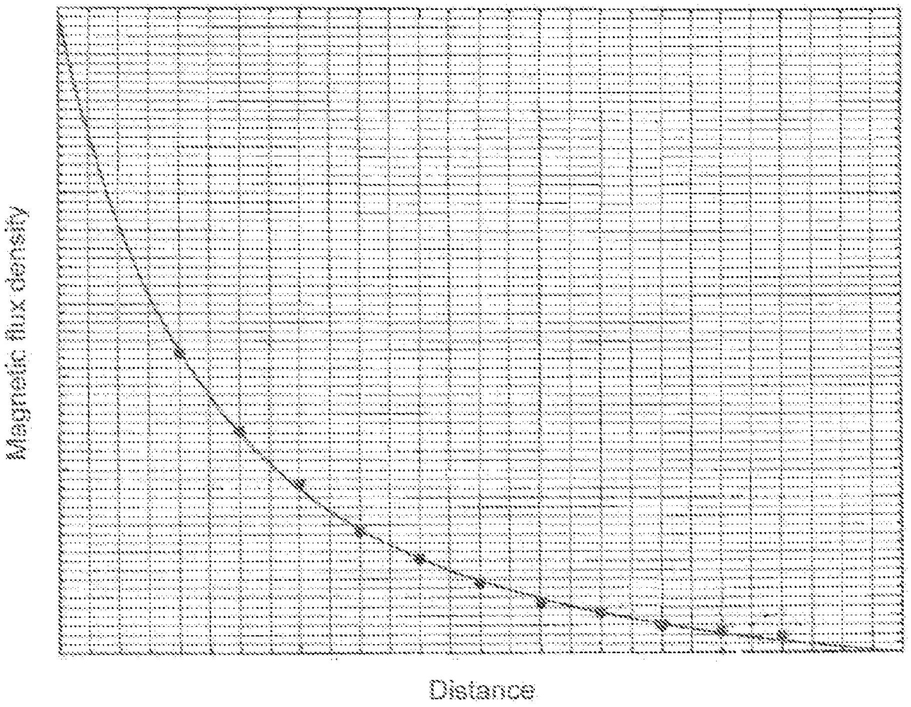

Fig. 18

MAGNETIC INTERFERENCE REDUCING SURGICAL DRAPE

CROSS-REFERENCE TO RELATED APPLICATIONS

The present application claims priority on U.S. Provisional Application No. 61/357,722, filed on Jun. 23, 2011, and No. 61/434,986, filed on Jan. 21, 2011, both incorporated herein by reference.

FIELD OF THE APPLICATION

The present application relates to the field of medical devices used during surgical procedures, interventions and treatments, and more particularly to a magnetic drape used in the surgical room, intervention room or treatment room for holding instruments in close vicinity to the treatment site on a patient.

BACKGROUND OF THE ART

Surgeons and medical professionals and personnel (e.g., doctors, veterinarians, dentist, nurses) use a plurality of instruments in performing surgery/treatment such as scalpels, forceps, sharps, scissors, clamps, needles, etc. The various instruments each have a specific function during surgery/treatment. Rigid trays are commonly used in order to support the plurality of instruments during surgery/treatment. The trays are typically kept horizontal by a stand or other structure so as to be relatively close to the surgical/treatment site. Medical personnel is often required to select and hand the instruments to the surgeon from the tray. Oftentimes, a nurse or the like is dedicated to selecting and handing instruments as requested by the surgeon.

It also has been common for medical personnel to place instruments directly on the patient surface for easy access to the instruments, and to create a transfer zone close to the treatment site. Such hands-free practice reduces the risk of percutaneous injury to medical personnel as well as the possibility of dropping the instruments as a result of being passed back and forth. However, as the patient usually represents an uneven surface, instruments may not be retained properly on the patient due to the effect of gravity and may roll or slide off the patient onto the floor.

A magnetic mat, magnetic drape or surgical drape (reusable in that it can be sterilized or single use) has been used into surgical rooms for a number of years as an alternative, in supplement or in combination to rigid trays for holding instruments or placing the instruments on the patient surface. One example thereof is the MagnaDrape™. The MagnaDrape™ consists of a flexible instrument-holding drape made of silicone, typically 12 inches by 16 inches, within which a plurality of permanent magnet units are positioned. The magnet units of the MagnaDrape™ are embedded or sandwiched in the drape. The flexible nature of the drape allows it to be laid onto uneven surfaces such as the body of the patient, in close proximity to the surgical or treatment site. The permanent magnet units of the MagnaDrape™ produce a magnetic field that will attract the surgical instruments and retain them on the drape even if the drape is not horizontal. As the permanent magnets are raised, there is defined free space between the permanent magnet units to facilitate the grasping of instruments. As they are typically made of ferromagnetic material, the surgical instruments do not have to be treated prior to being used with the MagnaDrape™ other than to be sterilized. Accordingly, with the MagnaDrape™, the surgical instruments may be retained in close proximity to or on a patient without the need for a support structure such as a stand.

However, the presence of permanent magnet units produces magnetic fields. The magnetic fields may have an effect on nearby electronic devices, implanted devices, or on the patient. For instance, a magnetic field of a given magnitude may alter the operation of implanted electronic devices used for stabilizing and/or controlling physiological parameters such as pacemakers, defibrillators, insulin pumps or regulators, pain control devices, any programmable medical devices or electronic devices, etc. Such magnetic fields can also affect the blood flow of the patient. In some instances, it is desired to reduce or avoid such side effect.

SUMMARY OF THE APPLICATION

It is an aim of the present disclosure to provide a magnetic drape that addresses issues associated with the prior art.

It is an aim of the present disclosure to provide a novel magnetic drape.

It is a further aim of the present disclosure to provide a magnetic drape producing a magnetic flux density of substantially lower magnitude on its support side than that on its exposed side so as to reduce magnetic interferences with implanted electronic devices.

It is a still further aim of the present disclosure to provide a magnetic drape producing a magnetic flux density of less than 10 Gauss on its support side.

Therefore, in accordance with the present application, there is provided a magnetic drape comprising a drape body made of a flexible material, the drape body having a panel portion having an undersurface adapted to be laid on an uneven body and a top surface, and a given thickness between the undersurface and the top surface; and a plurality of magnet units within the flexible material of the drape body, with each said magnet unit comprising a shielding material in the shape of a cup oriented to have a bottom wall facing toward the undersurface of the drape body, and at least one magnet received at least partially in the cup, the at least one magnet comprising at least two sections arranged to expose opposite polarity on a top surface of the magnetic drape, with each said section having opposite polarities oriented vertically.

Further in accordance with the present disclosure, the magnet units each have any one of a circular shape and a rectangular shape.

Still further in accordance with the present disclosure, the cups each have a circular shape, and further comprising two sections of magnets in each said cup, each said section having a semi-circular shape.

Still further in accordance with the present disclosure, the cups each have a circular shape, and further comprising four sections of magnets in each said cup, each said section having a quarter shape, with the sections exposing a N-S-N-S sequence of opposite poles in the top surface of the magnet unit.

Still further in accordance with the present disclosure, the cups each have a circular shape, and further comprising six sections of magnets in each said cup, each said section having a sixth shape, with the sections exposing a N-S-N-S-N-S sequence of opposite poles in the top surface of the magnet unit.

Still further in accordance with the present disclosure, the cups each have a circular shape, and further comprising eight said sections in each said cup, with the sections exposing a N-S-N-S-N-S-N-S sequence of opposite poles in the top surface of the magnet unit.

Still further in accordance with the present disclosure, the cups each have a circular shape, and further wherein the at least one magnet comprises three sections in each said cup, each said section having an elongated shape with the sections arranged side by side to define a circular shape, with the magnets exposing a sequence of opposite poles in the top surface of the magnet unit.

Still further in accordance with the present disclosure, the cups each have a circular shape, and further wherein the at least one magnet comprises three sections in each said cup, each said section having an elongated shape with the sections arranged side by side to define a circular shape, with lateral sections exposing opposite poles in the top surface of the magnet unit, and a central section being oriented relative to the lateral sections to create a Halbach effect.

Still further in accordance with the present disclosure, the cups each have a rectangular shape, and wherein the at least one magnet comprises two sections in each said cup, each said section having an elongated shape with the sections arranged side by side to define a rectangular shape.

Still further in accordance with the present disclosure, the cups each have a rectangular shape, and wherein the at least one magnet comprises two sections in each said cup, each said section having a square shape with the sections arranged side by side to define a rectangular shape.

Still further in accordance with the present disclosure, the cups each have a rectangular shape, and wherein the at least one magnet comprises four sections in each said cup, each said section having a square or rectangular shape with the sections arranged side by side to define a rectangular shape.

Still further in accordance with the present disclosure, the cups each have a circular shape with at least two cavities, and further comprising one of said sections of magnet in each said cavity of the cups.

Still further in accordance with the present disclosure, the cavities have one of a circular shape and a triangular shape.

Still further in accordance with the present disclosure, shielding means are connected to or incorporated in the drape body below the plurality of magnet units.

Still further in accordance with the present disclosure, the shielding means is at least one of a shielding sheet, a shielding mesh, a shielding material, and shielding particles.

Still further in accordance with the present disclosure, at least one of a fluorescent and phosphorescent coating or additive is on the top surface of the drape body.

Still further in accordance with the present disclosure, the magnet units project upwardly from the top surface of the panel portion of the drape body.

Still further in accordance with the present disclosure, the given thickness of the panel portion is at least ⅛ in such that a magnetic flux density at the undersurface of the drape body is less than 10 Gauss.

Still further in accordance with the present disclosure, the given thickness of the panel portion is sized to keep the magnet units at a distance of at least ⅛ in from an electronic device in a patient when the magnetic drape is laid on the patient, such that a magnetic flux density is less than 10 Gauss at the electronic device.

BRIEF DESCRIPTION OF DRAWINGS

FIG. **1** is a top plan view, segmented, of a magnetic drape in accordance with an embodiment of the present disclosure;

FIG. **2** is a schematic side elevation view of the magnetic drape of FIG. **1**;

FIG. **3** is a schematic side view of a magnetic drape in accordance with another embodiment of the present disclosure, with longitudinal channels in an undersurface thereof;

FIG. **4** is a schematic side elevation view of a magnetic drape with a shielding sheet or mesh sheet in accordance with another embodiment of the present disclosure;

FIGS. **5**A and **5**B are respectively a schematic top view and side view of a magnet unit in accordance with an embodiment of the present disclosure, the magnet unit having a circular single permanent magnet suitably oriented and encapsulated in a metallic cup;

FIGS. **6**A and **6**B are schematic top and side views of the magnet unit of FIGS. **5**A and **5**B, featuring hemi-spherical sections exposing opposed poles at the top of a cup; and

FIG. **7** is a schematic top view of the magnet unit of FIG. **5**A, featuring elongated sections exposing opposed poles at the top of the cup;

FIG. **8** is a schematic top view of the magnet unit of FIG. **5**A, featuring quarter-shaped sections exposing opposed poles at the top of the cup;

FIG. **9** is a schematic top view of the magnet unit of FIG. **5**A, featuring sixth-shaped sections exposing opposed poles at the top of the cup;

FIG. **10** is a schematic top view of the magnet unit of FIG. **5**A, featuring eighth-shaped sections exposing opposed poles at the top of the cup;

FIGS. **11**A and **11**B are schematic top and side views of the magnet unit of FIG. **5**A, featuring elongated lateral sections exposing opposed poles at the top of the cup, with a central section creating a Halbach effect;

FIGS. **12**A and **12**B are respectively a schematic top view and side view of a magnet unit used in accordance with an embodiment of the present disclosure, the magnet unit having a rectangular single permanent magnet suitably oriented and encapsulated in a metallic cup;

FIG. **13** is a schematic top view of the magnet unit of FIGS. **12**A and **12**B, featuring elongated sections exposing opposed poles at the top of the cup;

FIGS. **14**A and **14**B are schematic top and side view of the magnet unit of FIGS. **12**A and **12**B, featuring rectangular sections exposing opposed poles at the top of the cup;

FIG. **15** is a schematic top view of the magnet unit of FIGS. **12**A and **12**B, featuring four rectangular sections exposing opposed poles at the top of the cup;

FIG. **16** is a schematic top view of a magnet unit having cavities with circular magnet sections exposing opposed poles at the top of the cup;

FIG. **17** is a schematic top view of a magnet unit having cavities with triangular magnet sections exposing opposed poles at the top of the cup; and

FIG. **18** is a graph illustrating a general trend of magnetic flux density as a function of a distance from a magnet.

DESCRIPTION OF PREFERRED EMBODIMENTS

Referring concurrently to FIGS. **1** and **2**, a magnetic drape in accordance with an embodiment of the present disclosure is generally shown at **10**. The magnetic drape **10** may be used to support instruments in surgery, treatment, dentistry, veterinary medicine, or in any other applications. For simplicity purposes, the description below will generally refer to the use of the magnetic drape **10** to support instruments in surgery, but it will be understood that the magnetic drape **10** may be used in other applications. Moreover, the term “drape” is used, but is not meant to be limitative. The drape is also know as a support mat, panel, etc.

The magnetic drape **10** has a drape body **12** with a top surface **14** and an undersurface **16**. The drape body **12** is made of a semirigid or flexible material and may also be known as a pad, a drape, a carpet or the like. Materials that may be used include nonrestrictively various medical-grade polymers and elastomers such as silicone, rubbers, etc., depending on the specifications of use of the magnetic drape **10** (e.g., reusable and sterilizable, single-use drape). The drape body **12** may have any appropriate dimensions to lie on the body of a patient and support instruments thereon. It is also considered to mold a mesh (e.g., nylon mesh) into or on the drape body **12** to alter its structural integrity, or to provide tear-resistance to the drape body **12**. However, the drape body **12** may be without such a nylon mesh.

When laid on the body of a patient, the drape body **12** conforms to the shape of the surface upon which it rests because of the flexible nature of its material. The drape body **12** is placed such that the undersurface **16** lies against the surface, whereas the top surface **14** is oriented upwardly.

Still referring to FIGS. **1** and **2**, magnet units **20** are raised from a panel portion of the drape body **12** and therefore define the top surface **14** of the drape body **12**, and are aligned in rows and columns. In another embodiment, the magnet units **20** are fully encapsulated in the material of the drape body **12**, whereby the top surface of the magnetic mat **10** is flat. The magnetic units **20** typically comprise a permanent magnet or permanent magnets that may be encapsulated, sandwiched or embedded in the material of the drape body **12**, and include a supporting shielding cup. The magnet units **20** are illustrated as being circular in FIGS. **1** and **2**, but may have any other appropriate shape, such as a rectangle, a strip or a bar as shown hereinafter. Moreover, the arrangement of components of the magnet units **20** is described in further details hereinafter.

Accordingly, the presence of the permanent magnet units **20** in accordance with the embodiment of FIGS. **1** and **2** will allow ferromagnetic instruments to be retained on the magnetic drape **10** when placed thereon by medical personnel or any technician. The magnetic forces of the magnet units **20** will pull the instruments to the magnetic drape **10**. The magnets of the magnet units **20** may for instance be ferrite ceramic magnets (e.g., anisotropic), among numerous possibilities, and may adopt any appropriate shape (e.g., circular, rectangular).

Referring to FIG. **2**, there is illustrated the magnetic drape **10** of FIG. **1**. It is observed that the thickness of the drape body **12**, namely between the top surface **14** and the undersurface **16**, is nonnegligible. It is known that the flux density of a magnetic field reduces in value as a function of a distance from the magnets. There is provided in FIG. **18** a graph illustrating a general trend of the magnetic flux density of the magnetic field as a function of a distance to a surface. Accordingly, by having the drape body **12** provided with a selected thickness, it is possible to have a minimal magnetic flux density under the undersurface **16**.

Referring to FIG. **3**, there is illustrated the drape body **12** with undersurface **30**. The undersurface **30** has a plurality of longitudinal channels **32**. The longitudinal channels **32** are provided so as to increase the distance between the top surface **14** and the undersurface **30** of the drape body **12**, in view of having a minimal magnetic flux density at the undersurface **30**, while increasing the flexibility of the mat **10**. As there is less material above the longitudinal channels **32**, the drape body **12** is more flexible. Alternative shapes are considered as well, such as posts, ribs or the like.

Referring to FIG. **4**, there is illustrated another embodiment of the magnetic drape **10**, in which a shielding sheet **40** is provided in the drape body **12**. The shielding sheet **40** may be laminated to the undersurface **16**, or may simply be molded into the drape body **12**. Although the shielding sheet **40** is illustrated as being closer to the undersurface **16** than the top surface **14**, the shielding sheet **40** may be at any suitable location in the drape body **12**. In such a case, the drape body **12** may consist of different layers bonded to one another, for instance to encapsulate shielding material therebetween. Alternatively, multiple layers of shielding sheets may also be used and as an example, one layer can be laminated closer to the undersurface while another can be closer to the top surface. Examples of materials used for the shielding sheet **40** include sheets of ferromagnetic material or of foil. It is for instance considered to use metal, numetal, powders, nanomaterials, nano-metallurgical materials, such as nano-crystalline ferromagnetic cladding, among other possibilities. For instance, some embodiments of the Nanovate™ platforms by Integran may be used. Also, a mesh of shielding material (e.g., Nanovate™) may also be used.

It is considered to apply a phosphorescent or fluorescent coating to the magnetic mat **10**, or to add a phosphorescent or fluorescent coating to the material of the drape body **12**. Considering that some types of surgery are performed in minimal lighting, a magnetic mat **10** being phosphorescent or fluorescent will be visible to the medical personnel, showing an outline of the instruments.

Now that various arrangements of the magnetic drape **10** have been described, the configuration of the magnet units **20** are set forth. The magnet units **20** are constituted of one or more magnets supported on a shielding material which can be made of many materials such metal, numetal, powders, nanomaterials, nano-metallurgical materials, such as nano-crystalline ferromagnetic cladding, among other possibilities. The various configurations are devised in order to produce a magnetic flux density of less than 10 Gauss at a given distance below the undersurface **16** of the magnetic drape **10**. According to an embodiment, the magnetic flux density is less than 10 Gauss at a distance of ⅛ in from a bottom surface of the magnet units **20**.

The various configurations comprise magnets made of any appropriate material. For instance, ceramic **8** or polymeric-based materials are well suited as a material for magnets for the magnet unit **20**, in view of the required flux density of the magnetic field under the magnetic drape **10**.

Referring to FIGS. **5**A and **5**B, a general arrangement of the magnet unit **20** is shown. The magnet unit **20** of FIG. **5**A and **5**B may be used in the magnetic drape **10** of FIGS. **1** to **4**. The magnet unit **20** has at least one permanent magnet **60** encapsulated partially in a cup **62**. The magnet **60** has at least a portion of its lateral surface and its bottom face within the cup **62**, while the upper face is exposed. The cup **62** is made from a shielding material, such as mild steel (e.g., 1010 steel, 1018 steel), or any other appropriate material, whereby the magnetic flux density of the magnetic field is reduced away from the bottom face of the magnet **60**. When the magnet unit **20** is positioned in the magnetic drape **10**, the bottom face of the magnet **60** (hidden in the cup **62**) is oriented toward the undersurface **16** (FIG. **1**) of the magnetic drape **10**.

By way of example, the cup **62** has a wall thickness ranging between 0.02-0.06 in (although other thicknesses are possible), with an outer diameter of 0.86 in, although other outer diameters are possible. Depending on the thickness of the wall of the cup **62**, the magnet **60** has a diameter ranging between 0.74-0.82 in, with the magnet **60** being snugly or force-fittingly received in the cup **62**. A suitable height for the

combination of the magnet **60** and cup **62** is 0.16 in, with the magnet **60** and cup **62** concurrently defining a top planar surface of the magnet unit **20**.

Referring to FIGS. **6**A and **6**B, a configuration of the magnet unit **20** is shown, using the specifications (e.g., dimensions and materials) set forth with the general arrangement of FIGS. **5**A and **5**B. In the configuration of FIGS. **6**A and **6**B, the cup **62** supports a bi-polar vertically magnetized magnet **60**, with the magnet **60** exposing opposite poles on the top surface of the magnet unit **20**, as illustrated by N and S. As observed in FIG. **6**B, the magnet **60** has two sections, with each section arranged to have a first pole oriented toward the top surface of the magnet unit **20**, and an opposite pole oriented toward the bottom surface of the magnet unit **20**.

It is pointed out that the vertical direction is between the top surface **14** and the undersurface **16**, but it not necessarily representative of the orientation in which the drape **10** will be used. The drape **10** may indeed be inclined, or even have a portion that is hanging vertically.

Further examples are provided, but in the various embodiments, the magnet **60** must have at least two sections of opposite poles exposed at the top surface of the magnet **60**, which poles are produced by way of a magnetization process. Alternatively, the sections may be physically independent magnets in a same cup **62**, with the independent magnets reproducing any one of the pole arrangements described herein. For simplicity purposes, both embodiments of a single magnet with multiple sections, or multiple independent magnets in a same cup **62** will be referred to as sections of magnets, with the sections having a specific polarity arrangement to produce a magnetic flux density in accordance with the present invention.

Referring to FIG. **7**, another configuration of the magnet unit **20** is shown. In the configuration of FIG. **7**, the cup **62** supports the magnet **60**, with the magnet **60** having three vertically magnetized sections of alternating opposite poles on the top surface (N-S-N) of the magnet unit **20**, as illustrated by N and S. In similar fashion to the magnet unit **20** observed in FIG. **6**B, each of the sections of the magnet **60** is arranged to have a first pole oriented toward the top surface of the magnet unit **20**, and an opposite pole oriented toward the bottom surface of the magnet unit **20**.

Referring to FIG. **8**, another configuration of the magnet unit **20** is shown. In the configuration of FIG. **8**, the cup **62** supports the magnet **60** having four quarter-shaped vertically magnetized sections exposing a sequence of alternating opposite poles (N-S-N-S) on the top surface of the magnet unit **20**, as illustrated by N and S. In similar fashion to the magnet unit **20** observed in FIG. **6**B, each of the sections of the magnets **60** is arranged to have a first pole oriented toward the top surface of the magnet unit **20**, and an opposite pole oriented toward the bottom surface of the magnet unit **20**.

Referring to FIG. **9**, another configuration of the magnet unit **20** is shown. In the configuration of FIG. **9**, the cup **62** supports the magnet **60** with sixth-shaped vertically magnetized sections, with the sixth-shaped sections of the magnets **60** exposing a sequence of alternating opposite poles (N-S-N-S-N-S) on the top surface of the magnet unit **20**, as illustrated by N and S. In similar fashion to the magnet unit **20** observed in FIG. **6**B, each of the sections of the magnet **60** is arranged to have a first pole oriented toward the top surface of the magnet unit **20**, and an opposite pole oriented toward the bottom surface of the magnet unit **20**.

Referring to FIG. **10**, another configuration of the magnet unit **20** is shown. In the configuration of FIG. **10**, the cup **62** supports the magnet **60** with eighth-shaped vertically magnetized sections, with the eighth-shaped segments exposing a sequence of alternating opposite poles (N-S-N-S-N-S-N-S) on the top surface of the magnet unit **20**, as illustrated by N and S. In similar fashion to the magnet unit **20** observed in FIG. **6**B, each of the segments **60** is arranged to have a first pole oriented toward the top surface of the magnet unit **20**, and an opposite pole oriented toward the bottom surface of the magnet unit **20**.

Referring to FIGS. **11**A and **11**B, another configuration of the magnet unit **20** is shown. In the configuration of FIGS. **11**A and **11**B, the cup **62** supports the magnet **60** having three magnetized segments in similar fashion to FIG. **7**. However, the configuration of FIGS. **11**A and **11**B differs from the configuration of FIG. **7**, in that the magnets **60** are in a striped Halbach pattern. In the Halbach pattern, the central section of the magnets **60** is oriented sideways to have both north and south poles exposed from the top surface of the magnet unit **20**. The north pole of the central section of the magnet **60** is adjacent to an exposed south pole of a lateral section of the magnets **60**, whereas the south pole of the central section of magnet **60** is adjacent to an exposed north pole of the other lateral section of the magnet **60**, as illustrated by N and S. As shown in FIG. **11**B, the lateral sections of magnets **60** are arranged to have a first pole oriented toward the top surface of the magnet unit **20**, and an opposite pole oriented toward the bottom surface of the magnet unit **20**.

Other configurations are possible for the magnet unit **20** with a circular shape. For instance, the magnets **60** may be in a tenth-shaped sections configuration, or may even feature more magnet sections.

Referring to FIGS. **12**A and **12**B, the magnet unit **20** has a rectangular, strip or bar shape as opposed to a circular one. A general arrangement of the rectangular magnet unit **20** is shown in FIGS. **12**A and **12**B. The magnet unit **20** of FIG. **12**A and **12**B may be used in the magnetic drape **10** of FIGS. **1** to **4**. The magnet unit **20** has at least one permanent magnet **60'** encapsulated partially in a cup **62'**. The single magnet **60'** must be magnetized so as to have at least two opposite poles in the top surface of the magnet **60'**.

The magnet **60'** has at least a portion of its side surfaces and its bottom face within the cup **62'**, while the upper face is exposed. The cup **62'** is made from a shielding material, such as mild steel (e.g., 1010 steel, 1018 steel), or any other appropriate shielding material, whereby the magnetic flux density of the magnetic field is reduced away from the bottom face of the magnet **60'**. When the magnet unit **20** of FIGS. **12**A and **12**B is positioned in the magnetic drape **10**, the bottom face of the magnet **60'** (hidden in the cup **62'**) is oriented toward the undersurface **16** (FIG. **1**) of the magnetic mat **10**.

By way of example, the cup **62'** has a wall thickness ranging between 0.02-0.06 in, with a length of 1.62 in and a width of 0.5 in, although other dimensions are possible. A suitable height for the combination of the magnet **60'** and cup **62'** is 0.1 in, although other dimensions are possible, with the magnet **60'** and cup **62'** concurrently defining a top planar surface of the magnet unit **20**.

Referring to FIG. **13**, a rectangular configuration of the magnet unit **20** is shown. In the configuration of FIG. **13**, the cup **62'** supports a pair of elongated sections of the magnet **60'**, with the elongated sections of the magnet **60'** exposing opposite poles on the top surface of the magnet unit **20**, as illustrated by N and S. Each of the sections of the magnet **60'** is arranged to have a first pole oriented toward the top surface of the magnet unit **20**, and an opposite pole oriented toward the bottom surface of the magnet unit **20**.

Referring to FIGS. **14**A and **14**B, another configuration of the magnet unit **20** is shown. In the configuration of FIG. **14**A and **14**B, the cup **62'** supports a pair of rectangular sections of

the magnet **60'**, with the rectangular sections of the magnet **60'** exposing opposite poles on the top surface of the magnet unit **20**, as illustrated by N and S. As shown in FIG. **14**B, each of the sections of the magnet **60'** is arranged to have a first pole oriented toward the top surface of the magnet unit **20**, and an opposite pole oriented toward the bottom surface of the magnet unit **20**.

Referring to FIG. **15**, another configuration of the magnet unit **20** is shown. In the configuration of FIG. **14**A and **14**B, the cup **62'** supports four square sections of the magnet **60'**, with the square sections of the magnet **60'** exposing alternating opposite poles (N-S-N-S) on the top surface of the magnet unit **20**, as illustrated by N and S. Each of the sections of the magnet **60'** is arranged to have a first pole oriented toward the top surface of the magnet unit **20**, and an opposite pole oriented toward the bottom surface of the magnet unit **20**.

Referring to FIGS. **16** and **17**, yet another embodiment of the magnet unit **20** is shown. The magnet unit **20** of FIGS. **16** and **17** may be used in the magnetic mat **10** of FIGS. **1** to **4**. The magnet unit **20** comprises a plurality of smaller permanents magnets **70**N and **70**S, with the affixed N indicating a North polarity while the affixed S indicates a South polarity. The magnets **70** are each received in a respective cavity in a metallic cup **72**. The magnets **70** each have their lateral portion and their bottom face within a respective cavity of the cup **72**, while the upper face is exposed. The metallic cup **72** is made from a shielding material, such as 1018 steel, or any other appropriate material, whereby the magnitude of the magnetic field is reduced away from the bottom face of the magnets **70**. When the magnet unit **20** of FIGS. **7**A and **7**B is positioned in the magnetic mat **10**, the bottom faces of the magnets **70** (hidden in the cup **72**) are oriented toward the undersurface **16** (FIG. **1**) of the magnetic mat **10**.

In FIG. **16**, the magnet unit **20** comprises six magnets **70**, although a magnet unit **20** may comprise more or less permanent magnets. Moreover, the magnets **70** are shown in an alternating polarity about the magnet unit **20**. The magnets **70** are also arranged with diametrically opposed polarities, although other arrangements may also be suitable.

Also, the magnets **70** are illustrated as being circular, but may have other geometries. For instance, a plurality of pie-shaped magnets **70** may share one common circular cavity in the cup **72**, as illustrated in FIG. **17**.

The permanent magnets **20**, **60** and **70** are made of any suitable magnetic material. One type of permanent magnet that may be used in the magnetic drape **10** is a standard or commercial ferrite magnets, although samarium-cobalt magnets (model 26002, Grade 24, with 2% samarium and 17% cobalt from Armstrong Magnetics Inc.) may also be used. As for the cups **60** and **70**, they may be machined, cast, molded, etc.

In any of the embodiments of FIGS. **1** to **17**, there may be provided on the undersurface **16** of the magnetic drape **10** a logo, an icon, instructions or the like, to indicate that the mat **10** is upside down. The mat **10** should be used with the magnets **20** facing upwardly. These instructions may also be on the top surface **14** to indicate that the mat **10** is correctly installed. In an embodiment, these instructions are printed directly on the surfaces of the magnetic drape **10**.

Referring to FIG. **18**, a graph illustrates a relation between a distance and a magnetic flux density. According to the graph, an increase in distance from the magnet units **20** (e.g., below the undersurface **16** of the drape **10**) results in a decrease in the magnetic flux density. Accordingly, the drape **10** may have an increased thickness under the magnet units **20** so as to ensure that the magnetic flux density is below a predetermined value. The afore-described embodiments allow the magnetic drape **10** to maintain a magnetic flux density of less than 10 Gauss at a distance of ⅛ in below the bottom surface of the magnet units **20**. If a lower magnetic flux density is required, the thickness of the panel of the drape body **12** may be increased.

The above-described embodiments may be for single-use magnetic drapes or for sterilizable magnetic drapes that will be reused. The embodiments described above may be used individually, or in combination where applicable.

The invention claimed is:

1. magnetic drape comprising:

a drape body made of a flexible material, the drape body having a panel portion having an undersurface adapted to be laid on an uneven body and a top surface, and a given thickness between the undersurface and the top surface; and

a plurality of magnet units within the flexible material of the drape body, with each said magnet unit comprising a shielding material in the shape of a cup oriented to have a bottom wall facing toward the undersurface of the drape body, and at least one magnet received at least partially in the cup, the at least one magnet comprising at least two sections arranged to expose opposite polarity on a top surface of the magnetic drape, with each said section having opposite polarities oriented vertically.

2. The magnetic drape according to claim **1**, wherein the magnet units each have any one of a circular shape and a rectangular shape.

3. The magnetic drape according to claim **2**, wherein the cups each have a circular shape, and further comprising two sections of magnets in each said cup, each said section having a semi-circular shape.

4. The magnetic drape according to claim **2**, wherein the cups each have a circular shape, and further comprising four sections of magnets in each said cup, each said section having a quarter shape, with the sections exposing a N-S-N-S sequence of opposite poles in the top surface of the magnet unit.

5. The magnetic drape according to claim **2**, wherein the cups each have a circular shape, and further comprising six sections of magnets in each said cup, each said section having a sixth shape, with the sections exposing a N-S-N-S-N-S sequence of opposite poles in the top surface of the magnet unit.

6. The magnetic drape according to claim **2**, wherein the cups each have a circular shape, and further comprising eight said sections in each said cup, with the sections exposing a N-S-N-S-N-S-N-S sequence of opposite poles in the top surface of the magnet unit.

7. The magnetic drape according to claim **2**, wherein the cups each have a circular shape, and further wherein the at least one magnet comprises three sections in each said cup, each said section having an elongated shape with the sections arranged side by side to define a circular shape, with the magnets exposing a sequence of opposite poles in the top surface of the magnet unit.

8. The magnetic drape according to claim **2**, wherein the cups each have a circular shape, and further wherein the at least one magnet comprises three sections in each said cup, each said section having an elongated shape with the sections arranged side by side to define a circular shape, with lateral sections exposing opposite poles in the top surface of the magnet unit, and a central section being oriented relative to the lateral sections to create a Halbach effect.

9. The magnetic drape according to claim **2**, wherein the cups each have a rectangular shape, and wherein the at least one magnet comprises two sections in each said cup, each

said section having an elongated shape with the sections arranged side by side to define a rectangular shape.

10. The magnetic drape according to claim **2**, wherein the cups each have a rectangular shape, and wherein the at least one magnet comprises two sections in each said cup, each said section having a square shape with the sections arranged side by side to define a rectangular shape.

11. The magnetic drape according to claim **2**, wherein the cups each have a rectangular shape, and wherein the at least one magnet comprises four sections in each said cup, each said section having a square or rectangular shape with the sections arranged side by side to define a rectangular shape.

12. The magnetic drape according to claim **2**, wherein the cups each have a circular shape with at least two cavities, and further comprising one of said sections of magnet in each said cavity of the cups.

13. The magnetic drape according to claim **12**, wherein the cavities have one of a circular shape and a triangular shape.

14. The magnetic drape according to claim **1**, wherein the magnet units project upwardly from the top surface of the panel portion of the drape body.

15. The magnetic drape according to claim **14**, wherein the given thickness of the panel portion is at least ⅛ in such that a magnetic flux density at the undersurface of the drape body is less than 10 Gauss.

16. The magnetic drape according to claim **14**, wherein the given thickness of the panel portion is sized to keep the magnet units at a distance of at least ⅛ in from an electronic device in a patient when the magnetic drape is laid on the patient, such that a magnetic flux density is less than 10 Gauss at the electronic device.

17. The magnetic drape according to claim **1**, further comprising shielding means connected to or incorporated in the drape body below the plurality of magnet units.

18. The magnetic drape according to claim **17**, wherein the shielding means is at least one of a shielding sheet, a shielding mesh, a shielding material, and shielding particles.

19. The magnetic drape according to claim **1**, further comprising at least one of a fluorescent and phosphorescent coating or additive on the top surface of the drape body.

* * * * *
